# Editorial: Re-emergence of neglected tropical diseases amid the COVID-19 pandemic: epidemiology, transmission, mitigation strategies, and recent advances in chemotherapy and vaccines

**DOI:** 10.3389/fphar.2023.1265803

**Published:** 2023-09-18

**Authors:** Ranjan K. Mohapatra, Venkataramana Kandi, Veronique Seidel, Ali A. Rabaan

**Affiliations:** ^1^ Department of Chemistry, Government College of Engineering, Keonjhar, Odisha, India; ^2^ Department of Microbiology, Prathima Institute of Medical Sciences, Karimnagar, Telangana, India; ^3^ Strathclyde Institute of Pharmacy and Biomedical Sciences, University of Strathclyde, Glasgow, United Kingdom; ^4^ Molecular Diagnostic Laboratory, Johns Hopkins Aramco Healthcare, Dhahran, Saudi Arabia; ^5^ College of Medicine, Alfaisal University, Riyadh, Saudi Arabia; ^6^ Department of Public Health and Nutrition, The University of Haripur, Haripur, Pakistan

**Keywords:** NTDs, neglected tropical diseases, epidemiology, transmission, mitigation strategy, vaccines

**FIGURE 1:**
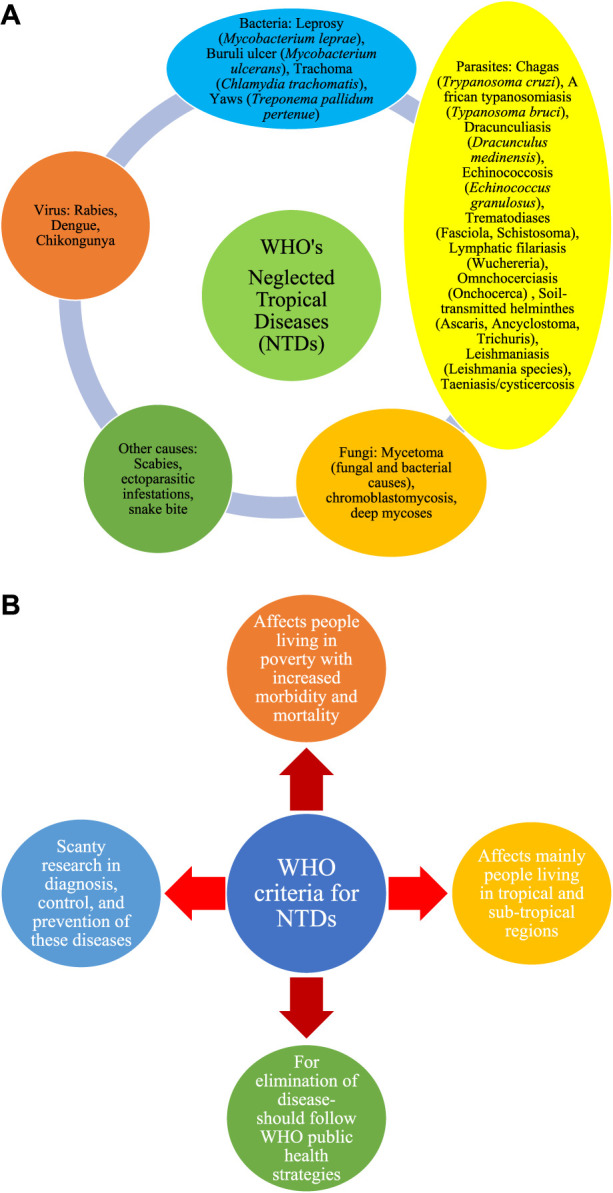
**(A)** List of NTDs as declared by WHO. **(B)** WHO criteria for NTDs.

Neglected tropical diseases (NTDs) are a group of infectious diseases that are common in the tropical regions of the world that include landmasses surrounding the equator such as North America, South America, Africa, Asia, and Australia. NTDs are caused by different microorganisms including bacteria, viruses, fungi, and parasites. Many NTDs involve specific environmental conditions, vectors, and animal reservoirs that favor the survival of microorganisms with complex life cycles. The vast majority of NTDs are caused by parasites followed by bacterial species, fungi, and viruses. Additionally, vector-borne arthropods like mites causing scabies and other ectoparasites can cause NTDs. A list of NTDs, as currently listed by the World Health Organization (WHO), is provided in Figure 1A.

During the 73rd World Health Assembly, the WHO proposed a road map for the elimination of NTDs by 2030. This mainly aims to control, prevent, and/or eliminate the WHO-listed NTDs (WHO, 2021). Given the emergence, and re-emergence, of novel and existing microbes, respectively, it is obvious that the list of NTDs proposed by the WHO is not exhaustive. Indeed, many other diseases may fall under the WHO criteria for NTDs (Figure 1B), including diseases affecting people living in poverty, and those residing in the tropical and sub-tropical areas of the world. In view of the rising incidence of NTDs and their influence on the social, economic, physiological, and psychological wellbeing of people, the WHO decided that 30 January 2022 be designated as the first World Neglected Tropical Diseases Day (WNTDD) (World Neglected Tropical Diseases Day, 2022).

During the COVID-19 pandemic, several cases of NTDs re-emerged in various countries worldwide. Mohapatra et al. reported a cluster of legionellosis (severe pneumonia) cases, including four deaths, in Argentina. *Legionella pneumophila* is a bacterium that commonly lives in the environment. In COVID-19 patients who suffered from debilitating post-disease syndromes, this opportunistic microbe was found to cause lung infections, especially in hospital settings, leading to nosocomial infections that further deteriorated the patients’ quality of life.

The ongoing COVID-19 pandemic not only contributed to the worsening of people’s health, but also affected health-related services that were in place for the management of existing diseases like tuberculosis (caused by *Mycobacterium tuberculosis*), malaria, dengue, measles, and diseases caused by the human immunodeficiency virus (HIV) among others. Additionally, preventive measures such as mass drug administration against filariasis and other parasitic infections were severely affected during the pandemic. Moreover, it was observed that the co-infection of COVID-19 among tuberculosis patients worsened health outcomes and contributed to an increase in morbidity and mortality (Satapathy et al.).

Further, Eastern equine encephalitis virus (EEEV) is a zoonotic virus belonging to the family Togaviridae and causes life-threatening encephalitis. It is a vector-borne/arboviral disease that is generally common in the equine population. There is evidence of accidental infections among humans and other vertebrate hosts. It has a high mortality rate, and more than half of infected persons suffer from the sequelae. The first human infection caused by EEEV was reported in Massachusetts, United States in 1938. Since then, sporadic cases of EEEV have been reported from various states confirming their existence in the environment and a re-emergence that accounted for a mortality rate greater than 40%. Despite being confined to the North American region, the virus may spread to non-endemic regions owing to cross-border animal transport and increased globalization (Sah et al.).

A re-emergence of the Marburg virus (MARV), the causative agent of viral hemorrhagic fever, was also noticed during the prevailing COVID-19 pandemic. MARV belongs to the same virus family group (Filovirus) as the Ebola virus. MARV is a highly pathogenic risk-group-4 virus that results in high mortality (approximately 90%) among infected persons. MARV infection has recently been reported in Ghana. Its re-emergence may be inevitable and could only be controlled with the discovery of an acceptable vaccine and therapeutic drugs which require further research (Islam et al.).

The recent outbreak of Ebolavirus (EBV) disease in Uganda appears to be the best example of how improved viral disease tracking capabilities allow health administrators and scientists to predict the spread and other clinical and epidemiological characteristic features (Branda et al.). This enables better preparedness among authorities, minimizes disease transmissibility, and contributes to better control.

Given that most diseases globally prevalent can be endemic to certain geographical regions, it is essential to understand their etiology, pathophysiology, epidemiology, diagnosis, management, control, and prevention. The same applies to SARS-CoV-2, which has been continuously mutating and evolving into several viral variants including the more pathogenic Delta and Omicron variants (Islam et al.). Therefore, public health administrators must use genomic surveillance methodologies to understand the viral evolution that could facilitate better preparedness to tackle future pandemic-like situations. Further, vaccine equity (availability to all, rich and poor) is an Research Topic that needs immediate attention along with improved vaccine that includes/covers recent variants of the virus and booster immunization doses for persons who are prone to re-infections (Rana et al.).

In a recent observation from Haryana, North India, hundreds of people reportedly presented to the hospitals with acute febrile illness (AFI). Later, diagnostic work-up among 58 of them revealed that the majority of patients were infected with Dengue virus (77.58%), followed by Chikungunya virus (3.44%), Japanese B Encephalitis virus (3.44%), and some had dual infections (2.23%). This study found none of the AFI patients had West Nile fever, scrub typhus, or leptospirosis. The results of this study emphasize the role of laboratory diagnostic methods in identifying the causative microbes during an outbreak or any similar health emergency (Satapathy et al.).

Despite being endemic to Africa, the Monkeypox virus (MPXV) was recently reported in non-endemic regions including the Americas, Europe, and other countries. Interestingly, the current MPXV outbreak was noted to be transmitted majorly through sexual routes. Additionally, the prevailing outbreak of MPXV has been noticed increasingly among HIV seropositive patients probably owing to their abnormal sexual activities (Yuan et al.). It was also observed that knowledge of MPXV was low (45%) among healthcare workers (HCWs). The vast majority (82%) of the HCWs believed that there is a need to learn more about the virus. Among the people who had suffered from COVID-19, many were afraid of MPXV compared to those who did not suffer from the disease (Swed et al.).

Among the various strategies that can be employed to tackle emerging, re-emerging, and other NTDs, vaccination and therapeutic drugs assume increased significance. A recent *in silico* study evaluated the efficacy of modified coptisine derivatives as a therapeutic alternative to treat infections with *Rhizomucor miehei* (fungus), *Mycolicibacterium smegmatis* (Mycobacteria), MPXV, and MARV (Akash et al.). Drugs that have been used to treat MPXV infection include tecovirimat, brincidofovir, cidofovir, vaccinia immune globulin, and trifluridine (Shamim et al.). Although not currently approved, there are two candidate vaccines in the pipeline against MPXV infection. This Research Topic provides up-to-date knowledge on the re-emergence of NTDs in the context of the COVID-19 pandemic and discusses how such diseases are transmitted and what mitigation strategies should be put in place to control their spread.
